# Lack of Anterior Communicating Artery Is Associated with Symptomatic Middle Cerebral Artery Atherosclerosis

**DOI:** 10.3390/biomedicines14051122

**Published:** 2026-05-15

**Authors:** Jia Li, Wenjie Yang, Lu Zheng, Xuelong Li, Winnie Chiuwing Chu, Thomas Waihong Leung, Xiangyan Chen

**Affiliations:** 1 Department of Neurology, Wuhan No. 1 Hospital, Wuhan 430022, China; jia-thomas.li@connect.polyu.hk; 2 Key Laboratory of Hubei Province for Neurological Disorders, Tongji Medical College, Huazhong University of Science and Technology, Wuhan 430030, China; 3 Department of Diagnostic Radiology and Nuclear Medicine, University of Maryland Baltimore, Baltimore, MD 21201, USA; yangwenjie.nju@gmail.com; 4 Department of Neurology, The Third Affiliated Hospital of Sun Yat-sen University, Guangzhou 510630, China; zhenglu6@mail.sysu.edu.cn; 5 Department of Neurology, The Second Affiliated Hospital of Guangzhou Medical University, Guangzhou 510260, China; xuelong.li@connect.polyu.hk; 6 Department of Imaging and Interventional Radiology, The Prince of Wales Hospital, The Chinese University of Hong Kong, Hong Kong SAR, China; winniechu@cuhk.edu.hk; 7 Division of Neurology, Department of Medicine and Therapeutics, The Prince of Wales Hospital, The Chinese University of Hong Kong, Hong Kong SAR, China; drtleung@cuhk.edu.hk; 8 Division of Science, Engineering and Health Studies, College of Professional and Continuing Education, The Hong Kong Polytechnic University, Hong Kong SAR, China

**Keywords:** acute ischemic stroke, middle cerebral artery atherosclerosis, cerebrovascular variation, anterior communicating artery

## Abstract

**Background**: Dysplasia or absence of anterior communicating artery (ACoA) is a common variation in the circle of Willis (COW) anomaly, and it may elevate the risks of cerebrovascular diseases. We aimed at investigating the association of ACoA dysplasia/absence with plaque imaging features of middle cerebral artery (MCA) atherosclerosis. **Methods**: We analyzed the prospective data from a vessel wall imaging cohort of adult patients suffering from acute ischemic stroke or transient ischemic attack due to intracranial atherosclerosis (2014 to 2020). Patients demonstrating MCA atherosclerotic plaques were included. The ACoA dysplasia/absence and other incomplete COW configurations were identified on magnetic resonance angiography. The MCA plaques were evaluated through high-resolution vessel wall imaging. **Results**: Of the 107 patients with MCA atherosclerosis, 29.9% showed ACoA dysplasia/absence. The patients with ACoA dysplasia/absence were more likely to have concomitant dysplasia/absence of anterior cerebral artery (71.9% vs. 18.7%, *p* < 0.001). For the 158 MCA plaques identified, those with ACoA dysplasia/absence exhibited a significantly higher prevalence of symptomatic status (58.7% vs. 31.3%, *p* = 0.001) and non-positive remodeling (58.7% vs. 26.8%, *p* < 0.001) than those without this variant. Regression analyses further demonstrated the robust association of ACoA dysplasia/absence with symptomatic status (odds ratio, 5.158; 95% confidence interval, 1.744–15.254; *p* = 0.003) and non-positive remodeling (odds ratio, 6.92; 95% confidence interval, 2.396–19.982; *p* < 0.001) of MCA atherosclerosis. **Conclusions**: As a common variation among patients with MCA atherosclerosis, ACoA dysplasia/absence may elevate the possibility to develop symptomatic MCA atherosclerosis, which showed non-positive remodeling. Stroke risk stratification based on the ACoA morphology needs further validation.

## 1. Introduction

Acute ischemic stroke (AIS) caused by intracranial atherosclerosis (ICAS) still burdens the society with a high risk of disability and mortality [1,2]. The middle cerebral artery (MCA) is the most prone to developing atherosclerosis among the major intracranial large arteries [3]. Previous studies described the geometric variations in the cerebral vasculature and their robust relevance to the progression of MCA atherosclerosis [4,5,6].

Dysplasia or absence of anterior communicating artery (ACoA) is a common anatomical variation in the circle of Willis (COW) anomaly among normal populations [7,8]. Growing evidence indicated the essential role of the ACoA in the cerebral hemodynamics, thereby affecting the development of cerebrovascular diseases [9,10]. However, whether the ACoA dysplasia or absence may have a clinical impact on the development of MCA atherosclerosis is rarely discussed.

In this study, we aimed at investigating the relationship between the anatomical pattern of the ACoA dysplasia or absence and plaque imaging features of MCA atherosclerosis among patient with AIS via high-resolution vessel wall imaging (HR-VWI).

## 2. Materials and Methods

### 2.1. Study Subject

**Figure 1:**
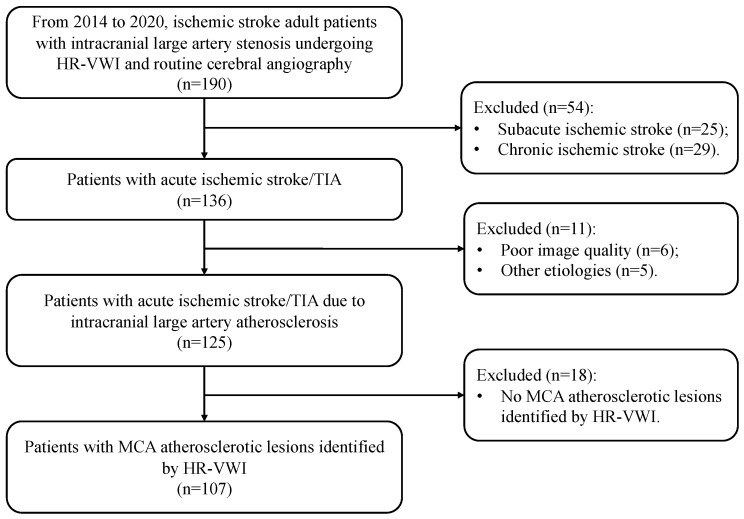
The flowchart indicates the patient selection in this study. HR-VWI, high-resolution vessel wall imaging; MCA, middle cerebral artery; TIA, transient ischemic attack.

We performed a retrospective review of the HR-VWI images obtained from consecutive adult patients presenting with AIS or transient ischemic attack attributable to ICAS. All patients were enrolled from the stroke center of the Prince of Wales Hospital between 2014 and 2020. HR-VWI was performed within seven days of initial stroke onset. Patients were eligible for inclusion if atherosclerotic plaques were identified within the MCA through HR-VWI. Patients were excluded if they met any of the following criteria: intracranial stenosis attributable to a non-atherosclerotic etiology, such as arterial dissection, vasculitis, or moyamoya disease; confirmed cardio-embolic source, including atrial fibrillation or valvular heart diseases; ipsilateral extracranial carotid stenosis exceeding 50%; a history of intracranial tumor, cerebrovascular malformation, or prior neurosurgical/endovascular intervention; or suboptimal image quality. The patient selection workflow was illustrated in Figure 1. Demographic data and traditional cardiovascular risk factors, including hyperlipidemia, hypertension, diabetes mellitus, and current smoking, were collected at the time of hospital admission. This study was conducted in accordance with the principles of the Declaration of Helsinki and received approval from the Joint Chinese University of Hong Kong-New Territories East Cluster Clinical Research Ethics Committee (No. 2015.011). Written informed consent was obtained from all participants or their designated family members.

### 2.2. Imaging Protocol

All examinations were performed on a 3.0 Tesla magnetic resonance imaging system (Achieva, Philips Healthcare, Amsterdam, The Netherlands) equipped with a dedicated 8-channel head coil. The imaging protocol comprised two primary sequences: a time-of-flight magnetic resonance angiography (TOF MRA) sequence centered at the COW, and a transverse 3-dimensional T1-weighted (T1w) Volumetric ISotropically Turbo spin echo Acquisition (VISTA) sequence spanning the intracranial vasculature from the V4 segment of the vertebral arteries to the distal MCA branches [5,11,12]. Pre-contrast VISTA imaging was followed by repeat acquisition approximately 5 min after intravenous administration of a gadolinium-based contrast agent (Dotarem, Gadoteric acid 0.5 mmol/mL, Guerbet, Roissy CdG Cedex, Villepinte, France) at a weight-adjusted dose of 0.1 mL/kg [5,11,12]. TOF MRA was acquired using the following parameters: field of view (FOV) of 200 × 200 × 56 mm^3^, acquired resolution of 0.4 × 0.6 × 0.7 mm^3^, repetition time (TR)/echo time (TE) of 23/3.5 ms, as well as scan duration of 3:07 min [5,11,12]. T1w VISTA was performed using the following parameters: FOV of 200 × 167 × 45 mm^3^, acquired resolution of 0.6 × 0.6 × 1.0 mm^3^, TR/TE 1500/36 ms, as well as scan duration of 6:51 min; the images were reconstructed to an isotropic spatial resolution of 0.5 × 0.5 × 0.5 mm^3^ [5,11,12].

### 2.3. ACoA Dysplasia/Absence and Other Incomplete COW Patterns

**Figure 2:**
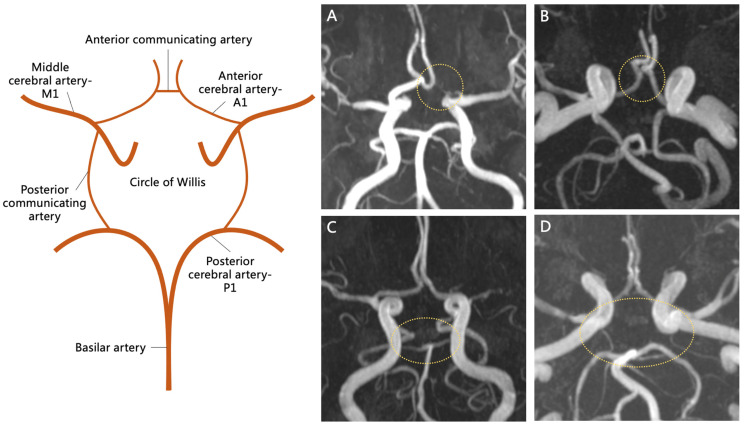
The geometric patterns of the circle of Willis. A simulated diagram shows the complete structure of the COW (**left**). TOF MRA images show dysplasia/absence of the anterior cerebral artery—A1 segment, the anterior communicating artery, the posterior cerebral artery—P1 segment, and the posterior communicating artery (**right** (**A**–**D**), yellow dashed outline). COW, circle of Willis; TOF MRA, time-of-flight magnetic resonance angiography (scale bar = 10mm).

TOF MRA imaging was employed to detect ACoA dysplasia or absence, as well as other incomplete COW patterns, including dysplasia or absence of the anterior cerebral artery (ACA) A1 segment, the posterior communicating artery (PCoA), and the posterior cerebral artery (PCA) P1 segment [5,13] (Figure 2). Dysplasia or absence of the intracranial artery was defined as a diameter of less than 0.8 mm on TOF MRA imaging [5,13].

### 2.4. Qualitative Assessment of MCA Atherosclerotic Plaques

**Figure 3:**
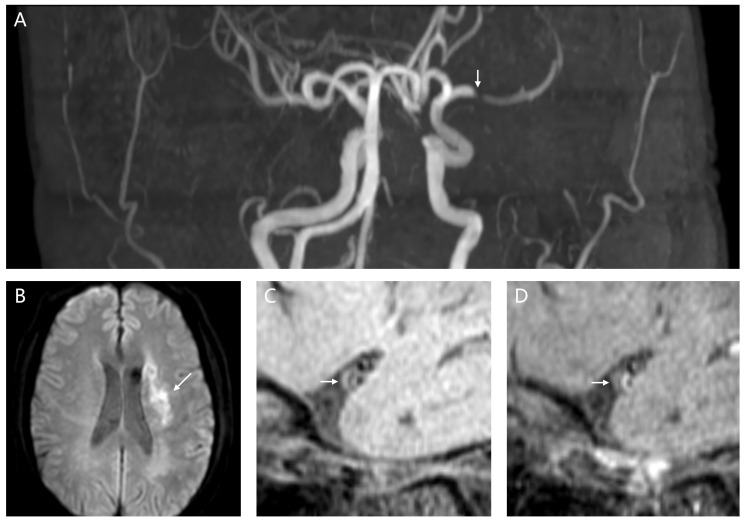
Identification of symptomatic MCA atherosclerotic plaque. (**A**) A symptomatic stenosis of the left MCA-M1 segment on magnetic resonance angiography imaging (white arrow). (**B**) Acute infarct in the left MCA-M1 territory on diffusion-weighted imaging (white arrow). (**C**,**D**) An eccentric, focal plaque in the left MCA-M1 segment as a symptomatic atherosclerotic lesion before and after administrating contrast on T1-weighted imaging, respectively (white arrows). MCA, middle cerebral artery (scale bar = 10 mm for (**A**,**B**); scale bar = 5 mm for (**C**,**D**)).

Atherosclerotic plaque was designated as focal vessel wall thickening on pre- and post-contrast T1w HR-VWI images [11,12]. Plaques involving the M1 and M2 segments of the MCA were evaluated (Figure 3). Pre-contrast plaque signal intensity (SI) was evaluated relative to adjacent normal gray matter parenchyma, with hyperintense and hypointense signal assessed separately for each plaque [11,12]. Intraplaque hemorrhage (IPH) was defined as plaque SI more than 150% of that of adjacent gray matter on pre-contrast T1w imaging [11,12].

Each MCA plaque was categorized as asymptomatic or symptomatic on the basis of its relationship to the territory of acute ischemic injury [12]. A plaque was designated as symptomatic when it represented either the sole lesion or the most stenotic lesion within the ipsilateral MCA territory of acute infarction [12]. A plaque was classified as asymptomatic when it resided outside the territory of acute ischemia, or when a more stenotic lesion was present within the same vascular territory [12].

### 2.5. Quantitative Measurements of MCA Atherosclerotic Plaques

Cross-sectional vessel wall measurements were obtained at the site of maximum luminal narrowing by reconstructing the short axis of each MCA lesion utilizing VesselMass software V2018-EXP-18/09/17 (Leiden University Medical Center, Leiden, The Netherlands) [11,12,14,15]. A morphologically normal segment proximal to the plaque was used as the reference; if unavailable, a normal-appearing distal segment or contralateral artery was selected [11,12].

Outer wall area (OWA) and lumen area (LA) were delineated by manually tracing of the vessel–cerebrospinal fluid interface and the blood–intimal interface, respectively [11,12]. The vessel wall area (VWA) was derived by subtracting LA from OWA [11,12]. The degree of luminal stenosis was computed as (1 − lesion LA/reference LA) × 100% [11,12]. Plaque burden was expressed as (lesion VWA/lesion OWA) × 100% [11]. The remodeling index was calculated as the ratio of lesion OWA to reference OWA; arterial remodeling was categorized as positive (remodeling index ≥ 1.05), intermediate (remodeling index = 0.95–1.05), and negative (remodeling index < 0.95) [11]. For subsequent analyses, intermediate and negative remodeling were grouped as non-positive remodeling [11].

For SI measurements, plaque SI on matched pre- and post-contrast T1w images was normalized to the SI of the adjacent gray matter, measured using a standardized circular region of interest (10 to 12 mm^2^) [11,12]. The plaque contrast enhancement index was defined as the percent change in normalized plaque SI between pre- and post-contrast images and was quantified as [(plaque SI/gray-matter SI) post − (plaque SI/gray-matter SI) pre]/(plaque SI/gray-matter SI) pre × 100% [11,12].

### 2.6. Statistical Analysis

All statistical analyses were performed using SPSS version 26.0 (IBM, Armonk, NY, USA). Categorical variables were reported as frequencies and proportions, and continuous variables were listed as medians with the interquartile range (IQR). Between-group comparisons of baseline clinical characteristics and plaque imaging features were conducted using Chi-squared test, Fisher’s exact test, or Mann–Whitney U test, as dictated by the nature and distribution of each variable. Univariate and multivariate logistic regression models were performed to examine the independent association between ACoA dysplasia or absence and MCA plaque imaging features. Multivariable models were adjusted for age, sex, and all covariates with a univariate *p* value lower than 0.1. Statistical significance was defined as a two-tailed *p* value lower than 0.05. Inter- and intra-observer reliability for the assessment of ACoA dysplasia or absence was evaluated using intraclass correlation coefficient with 95% confidence interval; excellence was defined as a coefficient over 0.81.

## 3. Results

### 3.1. Patient Baseline Characteristics and ACoA Dysplasia/Absence

This study included 107 patients with MCA atherosclerotic plaques (median age, 63 years old; 62.6% male). Baseline demographic and clinical features of these patients were showed in Table 1. The prevalence of dysplastic or absent ACoA was 29.9%. Notably, patients with dysplastic or absent ACoA exhibited a significantly higher prevalence of ACA dysplasia or absence, compared to those with a normal ACoA (71.9% vs. 18.7%; *p* < 0.001). However, there were no statistically significant differences in other baseline characteristics between the two groups (all *p* values > 0.05).

### 3.2. MCA Atherosclerosis and ACoA Dysplasia/Absence

A total of 158 MCA atherosclerotic lesions were identified through HR-VWI. The comparisons of MCA plaque imaging characteristics depending on the presence of normal vs. dysplastic or absent ACoA are displayed in Table 2. MCA plaques with dysplastic or absent ACoA were more possible to show symptomatic status than those with a normal ACoA (58.7% vs. 31.3%; *p* = 0.001). Furthermore, MCA plaques in the dysplastic or absent ACoA group demonstrated a significantly higher prevalence of non-positive remodeling pattern, compared to those in the normal ACoA group (58.7% vs. 26.8%; *p* < 0.001). Yet, no significant differences in other plaque imaging features were identified between the two groups (all *p* values > 0.05).

### 3.3. Univariate and Multivariate Regression

As summarized in Table 3, the ACoA dysplasia or absence was significantly associated with symptomatic MCA plaques (odds ratio, 3.126; 95% confidence interval, 1.537–6.359; *p* = 0.002) and non-positive remodeling MCA plaques (odds ratio, 3.884; 95% confidence interval, 1.889–7.985; *p* < 0.001) in the univariate logistic regression models. Multivariate logistic regression models also demonstrated the independent relevance of ACoA dysplasia or absence to symptomatic status (odds ratio, 5.158; 95% confidence interval, 1.744–15.254; *p* = 0.003) and non-positive remodeling pattern (odds ratio, 6.92; 95% confidence interval, 2.396–19.982; *p* < 0.001) of MCA atherosclerosis, after adjustment for age, sex, dysplasia or absence of ACA, plaque hyperintensity signal, arterial remodeling pattern, and symptomatic status.

### 3.4. Inter- and Intra-Observer Reliability

Inter- and intra-observer reliability for assessing ACoA dysplasia or absence was excellent (coefficient = 0.923, 95% confidence interval 0.869–0.955; coefficient = 0.961, 95% confidence interval 0.934–0.978, respectively). Inter- and intra-observer reliability for evaluating plaque imaging characteristics has been previously reported as substantial to excellent [16,17].

## 4. Discussion

In the current study, we identified a high prevalence of the ACoA dysplasia or absence (29.9%) among patients with MCA atherosclerosis. This anatomical variant was revealed in independent correlation with increased risks of occurring both symptomatic presentation and non-positive remodeling pattern in MCA atherosclerosis.

Utilizing the TOF MRA imaging technique, a high rate of anatomical anomaly of the ACoA was detected in patients with MCA atherosclerosis. Our finding showed consistency in the prior studies which reported the prevalence of the ACoA variant morphology ranging between 7.5% and 32.7% [18,19,20]. We speculated that inter-study differences in population characteristics (e.g., age distribution, ethnicity, and cerebrovascular disease profiles) or imaging protocols might largely account for the observed fluctuation in the rates of the ACoA anomaly [21]. Importantly, the ACoA plays a pivotal role in cerebral collateral circulation and hemodynamic balance [9,22], the lack of which is considered as a hemodynamic perturbation mechanistically implicated in the pathogenesis of cerebrovascular diseases [10,23]. Therefore, the higher frequency of the ACoA anomaly in this cohort may suggest a potential contribution to MCA hemodynamic conditions that favor the development of MCA atherosclerotic plaques.

Prior studies on AIS due to large vessel occlusion have demonstrated that the ACoA represents a key conduit for cross-hemispheric anterior circulation collateralization, while lacking ACoA is associated with reduced collateral recruitment and poorer perfusion of distal territories [24,25,26]. Similarly, a patent ACoA provides collateral flow to the ipsilateral ACA in the event of MCA narrowing [27]. Cortical branches of the ipsilateral ACA then collaterally perfuse the distal MCA territory [27,28,29]. Under such circumstances, the ACoA collateral flow may largely increase blood flow in the MCA, thereby deferring ischemic progression within the penumbra region [27,28,30]. In contrast, reduced collateral function owing to a lack of the ACoA may contribute to chronic low/oscillatory cerebral blood flow in the MCA region and increase susceptibility to symptomatic plaque presentation [31]. This may explain the observed association between the ACoA variant morphology and symptomatic MAC plaque status in our study.

We also observed that the ACoA anatomical variation robustly increased the risk of occurring non-positive remodeling MCA atherosclerotic lesions. Positive remodeling enables intracranial arterial wall to expand outward in reaction to plaque progression, not influencing the actual lumen area; in contrast, negative remodeling engages the arterial wall in thickening inward [32]. Guo et al. recently demonstrated that progressive ICAS plaques were characterized by inward remodeling and concomitant lumen reduction [33]. As discussed above, reduced blood flow in the MCA attributed to the ACoA variation might alter local hemodynamic forces (e.g., lowering wall shear stress or promoting oscillatory blood flow) on the arterial wall and affect MCA remodeling capacity, thereby accelerating MCA atherosclerosis progression [31,34,35,36]. On the other hand, Yang et al. indicated that favorable collateral in posterior circulation might decrease the risk of arterial positive remodeling among patients with severe symptomatic vertebrobasilar atherosclerosis [37], which did not support our observation. We suspected that arterial remodeling differentiation between anterior and posterior circulations driven by hemodynamics, genetics, and sympathetic vascular innervation might account for the phenomenon [38,39].

Our findings have underlying clinical significance. The collateral flow provided by the ACoA was found to favor alleviation of neurovascular injury and improvement of neurobehavioral outcomes [40]. The presence of the ACoA may then function as a potential biomarker for predicting good clinical outcomes of revascularization in patients with anterior circulation infarction [27]. Meanwhile, we reported the robust association of the ACoA variation with MCA plaque imaging features. These observations provide a potential anatomical explanation for inter-individual variability in the clinical expression of MCA atherosclerosis and may help refine imaging-based characterization of plaque vulnerability. Accordingly, stroke risk stratification depending on the ACoA variant morphology may facilitate early identification and precise management of patients with acute stroke harboring MCA vulnerable plaques. Further prospective studies are needed to clarify their clinical significance.

Limitations of this study included: (1) Due to the observational, cross-sectional nature of the study, the conclusions should be drawn cautiously about proposing causality between ACoA dysplasia or absence and MCA plaque behavior. However, our findings provide a rationale for future mechanistic and clinical studies. (2) Our findings should not be generalized to other populations (e.g., carotid atherosclerotic stenosis or asymptomatic individuals). In addition, our results were restricted to the MCA plaques. Further investigation is warranted to characterize the impact of the ACoA dysplasia or absence on vulnerable atherosclerotic plaques within cerebral anterior circulation.

## 5. Conclusions

ACoA dysplasia or absence is a common anatomical variation among patients with MCA atherosclerosis, and it is independently correlated with increased risks of developing symptomatic and non-positive remodeling MCA atherosclerosis. These findings suggest a potential link between the COW morphology and MCA plaque vulnerability. Prospective validation in independent cohorts is required to confirm this association and clarify its clinical significance.

## Figures and Tables

**Table 1:** Demographic and clinical characteristics of subjects with MCA atherosclerosis between normal and dysplastic/absent ACoA.

Parameters	All Patients (*n* = 107)	Patients with Normal ACoA (*n* = 75)	Patients with Dysplastic/Absent ACoA (*n* = 32)	*p *Value
Age, years, median (IQR)	63 (55–71)	63 (52–70)	62 (55.5–71.75)	0.816
Male/female, *n*	67/40	45/30	22/10	0.392
Hypertension, *n* (%)	80 (74.8%)	54 (72%)	26 (81.3%)	0.313
Hyperlipidemia, *n* (%)	62 (57.9%)	42 (56%)	20 (62.5%)	0.533
Diabetes, *n* (%)	35 (32.7%)	26 (34.7%)	9 (28.1%)	0.509
Current smoking status, *n* (%)	28 (26.2%)	17 (22.7%)	11 (34.4%)	0.207
Index event				1
Stroke, *n* (%)	93 (86.9%)	65 (86.7%)	28 (87.5%)	
TIA, *n* (%)	14 (13.1%)	10 (13.3%)	4 (12.5%)	
Incomplete COW patterns				
ACA dysplasia/absence, *n* (%)	37 (34.6%)	14 (18.7%)	23 (71.9%)	<0.001
ACoA dysplasia/absence, *n* (%)	32 (29.9%)	-	-	
PCA dysplasia/absence, *n* (%)	29 (27.1%)	19 (25.3%)	10 (31.3%)	0.528
PCoA dysplasia/absence, *n* (%)	86 (80.4%)	62 (82.7%)	24 (75%)	0.361

ACA, anterior cerebral artery; ACoA, anterior communicating artery; COW, circle of Willis; IQR, interquartile range; MCA, middle cerebral artery; PCA, posterior cerebral artery; PCoA, posterior communicating artery; TIA, transient ischemic attack.

**Table 2:** Plaque imaging features of MCA atherosclerosis between normal and dysplastic/absent ACoA.

Characters	Lesions with Normal ACoA (*n* = 112)	Lesions with Dysplastic/Absent ACoA (*n* = 46)	*p *Value
Symptomatic status, *n* (%)	35 (31.3%)	27 (58.7%)	0.001
Hypointensity signal, *n* (%)	29 (25.9%)	11 (23.9%)	0.795
Hyperintensity signal, *n* (%)	49 (43.8%)	27 (58.7%)	0.088
IPH, *n* (%)	19 (17%)	12 (26.1%)	0.19
Arterial remodeling pattern			<0.001
Positive, *n* (%)	82 (73.2%)	19 (41.3%)	
Non-positive, *n* (%)	30 (26.8%)	27 (58.7%)	
Luminal stenosis, %, median (IQR)	68.89 (46.75–81.81)	70.25 (51.04–81.75)	0.726
Plaque burden, %, median (IQR)	81.34 (72.79–87.79)	82.72 (73.51–88.3)	0.484
Enhancement index, %, median (IQR)	18.41 (6.29–40.2)	19.51 (6.11–39.54)	0.726

ACoA, anterior communicating artery; MCA, middle cerebral artery; IPH, intraplaque hemorrhage; IQR, interquartile range.

**Table 3:** Regression models for independent association between MCA plaque imaging features and the dysplasia/absence of ACoA.

	Univariate Regression		Multivariate Regression *	
	OR 95% CI	*p* Value	OR 95% CI	*p* Value
Asymptomatic status	1 (ref.)		1 (ref.)	
Symptomatic status	3.126 (1.537–6.359)	0.002	5.158 (1.744–15.254)	0.003
Positive arterial remodeling	1 (ref.)		1 (ref.)	
Non-positive arterial remodeling	3.884 (1.889–7.985)	<0.001	6.92 (2.396–19.982)	<0.001

ACoA, anterior communicating artery; CI, confidence interval; MCA, middle cerebral artery; OR, odds ratio. * Adjusted for age, sex, dysplasia/absence of anterior cerebral artery, hyperintensity signal, arterial remodeling, and symptomatic status.

## Data Availability

The original contributions presented in this study are included in the article. Further inquiries can be directed to the corresponding author.

## Abbreviations

The following abbreviations are used in this manuscript:

ACA	anterior cerebral artery
ACoA	anterior communicating artery
AIS	acute ischemic stroke
COW	circle of Willis
FOV	field of view
HR-VWI	high-resolution vessel wall imaging
IQR	interquartile range
ICAS	intracranial atherosclerosis
IPH	intraplaque hemorrhage
LA	lumen area
MCA	middle cerebral artery
OWA	outer wall area
PCA	posterior cerebral artery
PCoA	posterior communicating artery
TE	echo time
TR	repetition time
SI	signal intensity
T1w	T1-weighted
TOF MRA	time-of-flight magnetic resonance angiography
VWA	vessel wall area
VISTA	Volumetric ISotropically Turbo spin echo Acquisition
